# Nitrogen and boron doped carbon layer coated multiwall carbon nanotubes as high performance anode materials for lithium ion batteries

**DOI:** 10.1038/s41598-021-85187-5

**Published:** 2021-03-11

**Authors:** Bo Liu, Xiaolei Sun, Zhongquan Liao, Xueyi Lu, Lin Zhang, Guang-Ping Hao

**Affiliations:** 1 Institute of Microbiology, Technische Universität Dresden, Zellescher Weg 20b, 01217 Dresden, Germany; 2 Tianjin Key Lab for Rare Earth Materials and Applications, Centre for Rare Earth and Inorganic Functional Materials, School of Materials Science and Engineering and National Institute for Advanced Materials, Nankai University, Tianjin, 300350 People’s Republic of China; 3 Fraunhofer Institute for Ceramic Technologies and Systems IKTS, Maria-Reiche-Strasse 2, 01109 Dresden, Germany; 4 Institute for Integrative Nanoscience, Leibniz Institute for Solid State and Materials Research (IFW Dresden), Helmholtzstrasse 20, 01069 Dresden, Germany; 5 Institut Für Festkörperphysik, Leibniz Universität Hannover, Appelstrasse 2, 30167 Hannover, Germany; 6 State Key Laboratory of Fine Chemicals, Liaoning Key Laboratory for Catalytic Conversion Carbon Resources, School of Chemical Engineering, Dalian University of Technology, Dalian, 116024 People’s Republic of China

**Keywords:** Energy storage, Materials science, Materials for energy and catalysis, Nanoscale materials

## Abstract

Lithium ion batteries (LIBs) are at present widely used as energy storage and conversion device in our daily life. However, due to the limited power density, the application of LIBs is still restricted in some areas such as commercial vehicles or heavy-duty trucks. An effective strategy to solve this problem is to increase energy density through the development of battery materials. At the same time, a stable long cycling battery is a great demand of environmental protection and industry. Herein we present our new materials, nitrogen and boron doped carbon layer coated multiwall carbon nanotubes (NBC@MWCNTs), which can be used as anodes for LIBs. The electrochemical results demonstrate that the designed NBC@MWCNTs electrode possesses high stable capacity over an ultra-long cycling lifespan (5000 cycles) and superior rate capability even at very high current density (67.5 A g^−1^). Such impressive lithium storage properties could be ascribed to the synergistic coupling effect of the distinctive structural features, the reduced diffusion length of lithium ions, more active sites generated by doped atoms for lithium storage, as well as the enhancement of the electrode structural integrity. Taken together, these results indicate that the N, B-doped carbon@MWCNTs materials may have great potential for applications in next-generation high performance rechargeable batteries.

## Introduction

With the world economic development, the demand for power requirements has increased rapidly, adding greatly to the greenhouse gases and the pollution problems mainly caused by the combustion of fossil fuels^1–3^. As a result, in recent years more efforts have been focused on renewable sources of electricity, which is any form of energy generated from natural resources (e.g. solar, wind, and biofuels)^4,5^. However, the instable productivity of these energy types remains a big challenge in many parts of the world. For instance, it is not practical for converting wind and solar power into continuous and stable electricity^6–8^. An ideal strategy is to store the generated electricity during the high productivity of the above-mentioned clean energy and release it gradually for the purpose of meeting the demand of electricity-consuming customers^9–12^. As a class of energy conversion and storage devices, rechargeable lithium ion batteries (LIBs) have many applied advantages such as high energy density, superior rate performance, and long cycling life, compared to other conventional batteries^13–15^. More recently, significant advances in LIBs have enabled their feasible use in stationary energy storage systems for solar and wind energy, and smart grids. Furthermore, they are also used widely as energy medium for various mobile devices and equipments like car, laptop, smart phone, and so on^16^. While, there are still some disadvantages in commercial LIBs, such as relatively high cost and low power density^17^. Thus, the usage of current LIBs is largely restricted to passenger cars and trucks, which require high efficiency and desired power output performance. Furthermore, it is well known that the high-power output is of immense importance for forcing vehicles to move fast, which is almost 4 times higher than of in moving status. To address these concerns, on the one hand engineers should pack more LIBs in series for achieving enough power and on the other hand, more advanced electrode materials enabling long cycle life, small size, reliable safety and low cost need to be fully exploited^18–21^.

Nowadays, in response to the environmental protection policy, it is important to develop electric trucks and buses, in the case that even more than 30% greenhouse gas emissions are caused by traditional vehicles or trucks, which are less than 4% of all vehicles in Europe^22^. Accordingly, most applications of these electric trucks and buses demand high-power performance that is fast charging and discharging capabilities. Nevertheless, it is also noted that the energy density of LIBs is higher than alkaline batteries, but is not high enough to use in vehicles, which can offer an all-electric range of 450 km^23^. Moreover, the battery could only serve the car for less than 8 years and with reduced capacity year after year, which may take high cost to customs. Notably, the cost of LIBs pack is between 50–80% of that of a whole car^24^. That means high costs and short life cycles are two other important factors to restrict the competitiveness of electric car in market. In particular, the charging time is another factor to limit the electric car development. Normally, an electric car needs roughly 5 h for charging, which has completely no competition with fossil car^23^. Hence, increasing the energy density, extending the stable cycling life, and enhancing the power density are essential to break through the dilemma of the application of LIBs not only in normal cars but also in trucks, buses and race cars. It has been demonstrated that increasing lithium ion storage ability of battery electrode is an effective way to improve the energy and power properties of LIBs^25–28^. Moreover, the enhanced current output can increase the power density directly, while simultaneous enhancement for all parameters remains big challenges.

Motivated from these facts, we hereby propose an effective strategy to prepare nitrogen and boron doped carbon layer coated multiwall carbon nanotubes (NBC@MWCNTs), which can act as high-performance anodes for lithium storage. The big challenge of this work is to coat porous carbon homogenously onto the MWCNT so that the whole material could possess very high wettability. The advance of this design is that electrolyte could easier diffuse into the materials to increase the active surface and to improve the electrochemical properties. Moreover, doping high amount of homogenic N/B in porous carbon is event more challengeable for the synthesis, which is rare to realize in previous research. In this work, the whole material forms a ball-block structure to save space. Furthermore, the presence of nanopores on the carbon layer can enhance the lithium ion transport ability and improve the lithium storage capacity^29–31^. In this design, it combines the inherent advantages of both CNT and N, B-doped carbons, which could ensure a fast electron transfer and high capacity. This feature makes it possible, that the current could transport directly through the whole CNT by an extreme high-power output and with high lithium ion storage capability^32,33^. The MWCNTs with numerous nanopores could also increase the capacity potential due to the increased reaction surface^29^. On the other side, carbon has low cost and stable cycle performance^34–36^. In addition, recent studies have also documented that the incorporation of heteroatoms (e.g., N, B, O) into nanostructured materials could enhance the transport kinetics during discharge/charge processes, which would enhance Coulombic efficiency and lithium capacity^37–40^. As a result, when used as anode materials for LIBs, the designed NBC@MWCNTs could exhibit high stable capacity over an ultra-long cycling lifespan (5000 cycles) and superior rate performance even at very high operational current density (67.5 A g^−1^).

## Results and discussion

**Figure 1:**
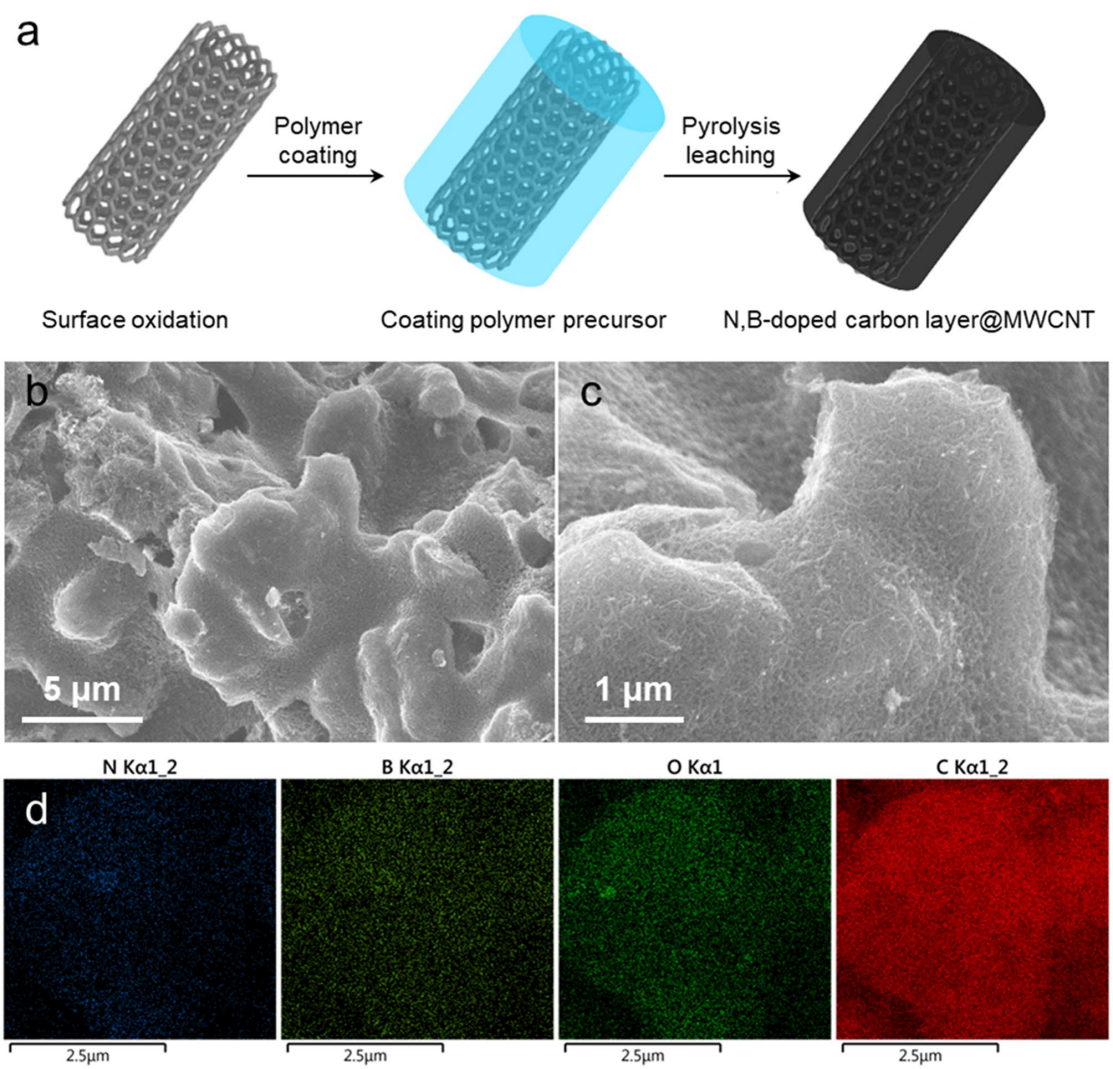
Structure and composition of NBC@MWCNTs. (**a**) The scheme of preparation procedure of NBC@MWCNTs; (**b**,**c**) SEM morphology of NBC@MWCNTs; (**d**) Elemental distribution mapping images of elements N, B, O, and C in NBC@MWCNTs products.

Figure 1a shows the synthetic route for the fabrication of the final products, which is also described in detail in the Experimental section. Briefly, MWCNTs were first oxidized and subsequently functionalized by a thin layer of metal–organic framework complex (4,4′-bipyridine-Cu). After pyrolysis and leaching process, the resultant MWCNTs featured with rich defects and heteroatoms (N, B, O) were then obtained. The surface morphology and microstructure of the fabricated NBC@MWCNTs were examined by scanning electron microscopy (SEM) with different magnifications, as displayed in Fig. 1b,c. Accordingly to the top morphology, it can be clearly seen that the tubular structure is still retained after coating thin carbon layer. All carbon nanotubes are highly aggregated and overlapped with each other with very thin wall, which can provide plenty of room for the improvement of lithium ion transport properties. As presented in Fig. 1d, the energy-dispersive X-ray spectroscopy (EDX) elemental mappings results further confirm the presence of N, B, C and O elements in the materials. It is observed that both N and B are uniformly distributed nanotubes, revealing the successful doping of N and B.

**Figure 2:**
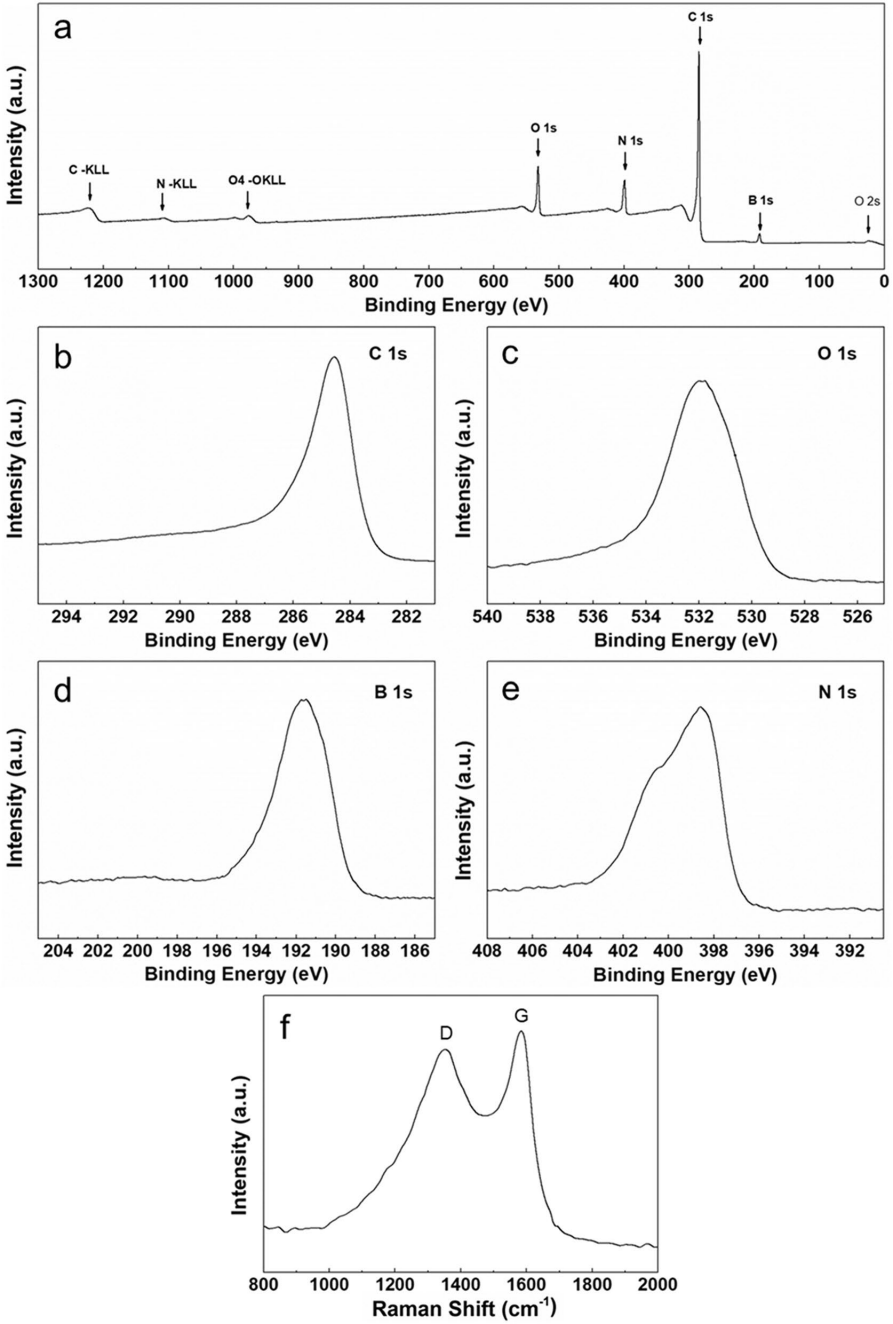
XPS and Raman spectra of NBC@MWCNTs. (**a**) survey spectrum; (**b**) C 1 s spectrum; (**c**) O 1 s spectrum; (**d**) B 1 s spectrum; (**e**) N1s spectrum, and (**f**) Raman spectrum of NBC@MWCNTs.

The nature of different elements shown in the materials was further determined by using X-ray photoelectron spectroscopy (XPS). As illustrated in Fig. 2a, no other peaks besides those for N, B, C and O can be seen in the wide survey spectrum, providing further evidence of the purity of this material. In Fig. 2b, the high-resolution of C 1 s, it can be observed that an intensive peak is exhibited at around 284.6 eV, suggesting high quantities carbon in the sample^37^. To compare with the main carbon peak, the high-resolution peaks located at 532.1 eV (Fig. 2c), 191.6 eV (Fig. 2d), 398.6 eV and 400.4 eV (Fig. 2e) are ascribed to O, B, and N at different microenvironments^41–43^. The quantitative estimation of different elemental species from the XPS peak intensity suggests the atomic percentage ratios for the NBC@MWCNTs are 73.73% for C 1 s, 9.79% for N 1 s, 8.60% for O 1 s and 7.88% for B 1 s, respectively. The high contents of N, B, and O containing species might serve as electrochemically active sites for making big contribution to the enhancement of lithium storage properties^44–47^.

Figure 2f displays the room temperature Raman spectrum of the sample for further investigation of the NBC@MWCNTs. Two characteristic carbon peaks are found at around 1351 and 1584 cm^−1^, which demonstrates the presence of carbon in the form of disordered structure. It is well known that the band around 1351 cm^−1^ (D band) with a lower intensity is related to the disordered carbon, and at 1584 cm^−1^ (G band) with a high intensity is assigned to graphitic carbon^35^. The latter peak corresponds to an E_2g_ mode of graphite, which is due to the sp^2^-bonded carbon atoms in a two-dimensional hexagonal graphitic layer^37^. In addition, it is interesting to note that the peak intensity ratio between the D and G bands (I_D_/I_G_) is ~ 0.9, demonstrating the partial crystallization of carbon species (CNT/carbon layer) during the coating process and high temperature thermal treatment.

**Figure 3:**
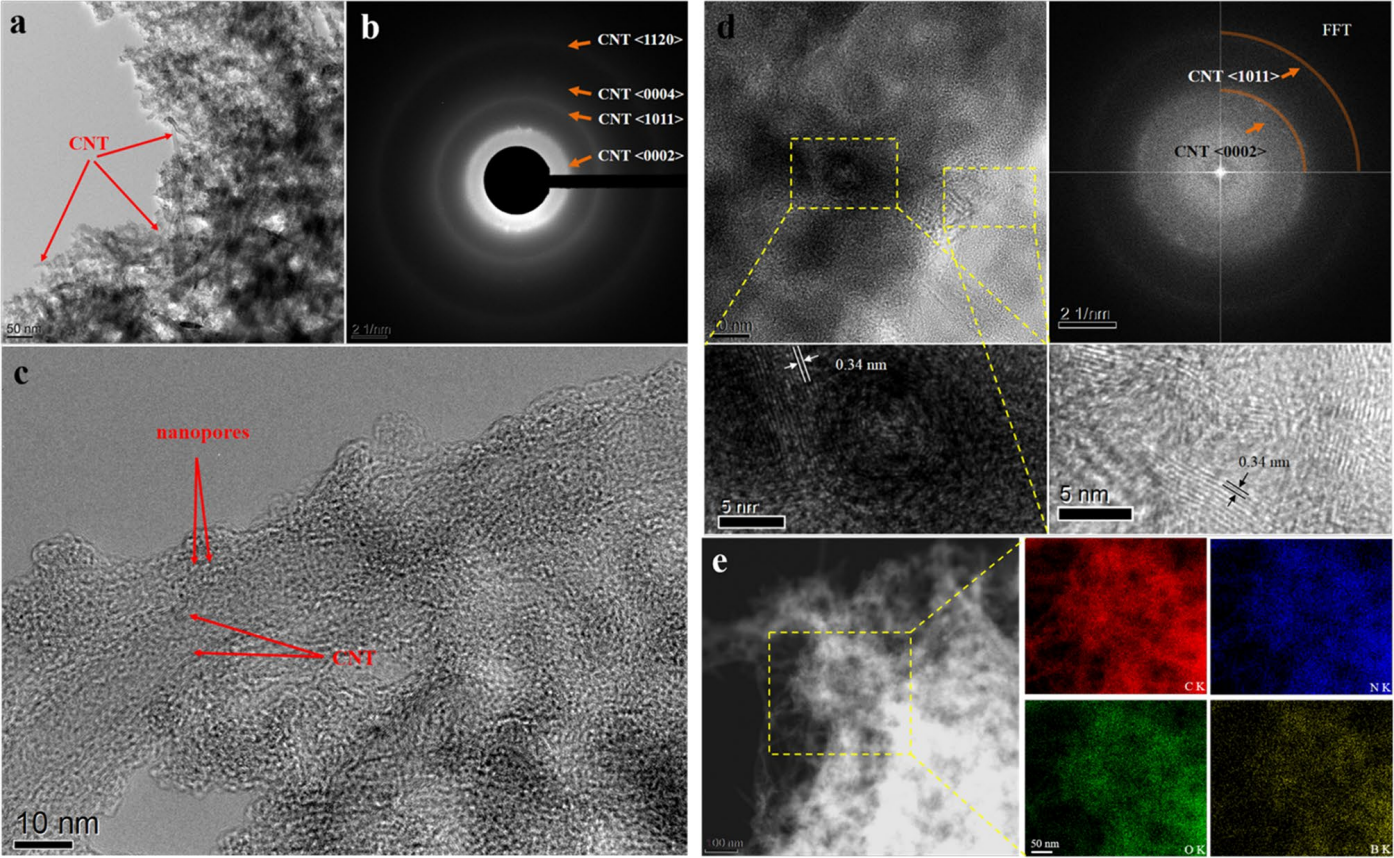
(**a**,**b**) low magnification TEM image and the corresponding SAED pattern of NBC@MWCNTs; (**c**) High resolution TEM image of a single CNT wrapped by nanoporous materials in NBC@MWCNTs; (**d**) High resolution TEM image and FFT pattern of the typical structure in NBC@MWCNTs; (**e**) HAADF image and corresponding elemental distribution mappings of N, B, O, and C in the NBC@MWCNTs.

Furthermore, transmission electron microscope (TEM) and high resolution TEM were utilized to carefully characterize the morphology and nanostructure of the obtained NBC@MWCNTs, and the results are displayed in Fig. 3. It can be clearly seen that the NBC@MWCNTs consist of homogeneously distributed CNTs wrapped by amorphous thin carbon layer marked with arrows in Fig. 3a. Besides, the corresponding selected area electron diffraction (SAED) pattern (Fig. 3b) shows a typical diffraction ring pattern that could be exactly identical to the coiled MWCNTs. The designed unique structure can be clearly revealed by a high resolution TEM image at the edge of the material (Fig. 3c). A MWCNT is coherently coated by a thin coating layer with some nanopores with a size of about 1 nm. The unique embedded structure is expected to be more beneficial for enhancing the lithium storage properties, and ensure the fast transportation of electrons inside the CNTs, when used as anodic materials in LIBs.

The typical high resolution TEM images in Fig. 3d (lower left and right) show common interplanar spacing of 0.34 nm for MWCNTs. Besides, the corresponding fast Fourier transform (FFT) pattern illustrated in the upper right also demonstrates the diffraction features of coiled MWCNTs (Fig. 3c). In order to further understand the nature of the product, high-angle annular dark-field scanning TEM (HAADF-STEM) was carried out. A typical HAADF-STEM image taken from the MWCNTs is displayed in Fig. 3e, where a few MWCNTs can be detected at the edge of the materials. The corresponding EDX elemental mappings performed on the same region indicated by the square (Fig. 3e) reveal that the designed materials are rich with carbon, nitrogen, boron, and oxygen. Taken together, it can be concluded that all four elements are coexisted and distributed homogenously in the matrix material, and nitrogen and boron are well doped onto the surface of MWCNTs.

**Figure 4:**
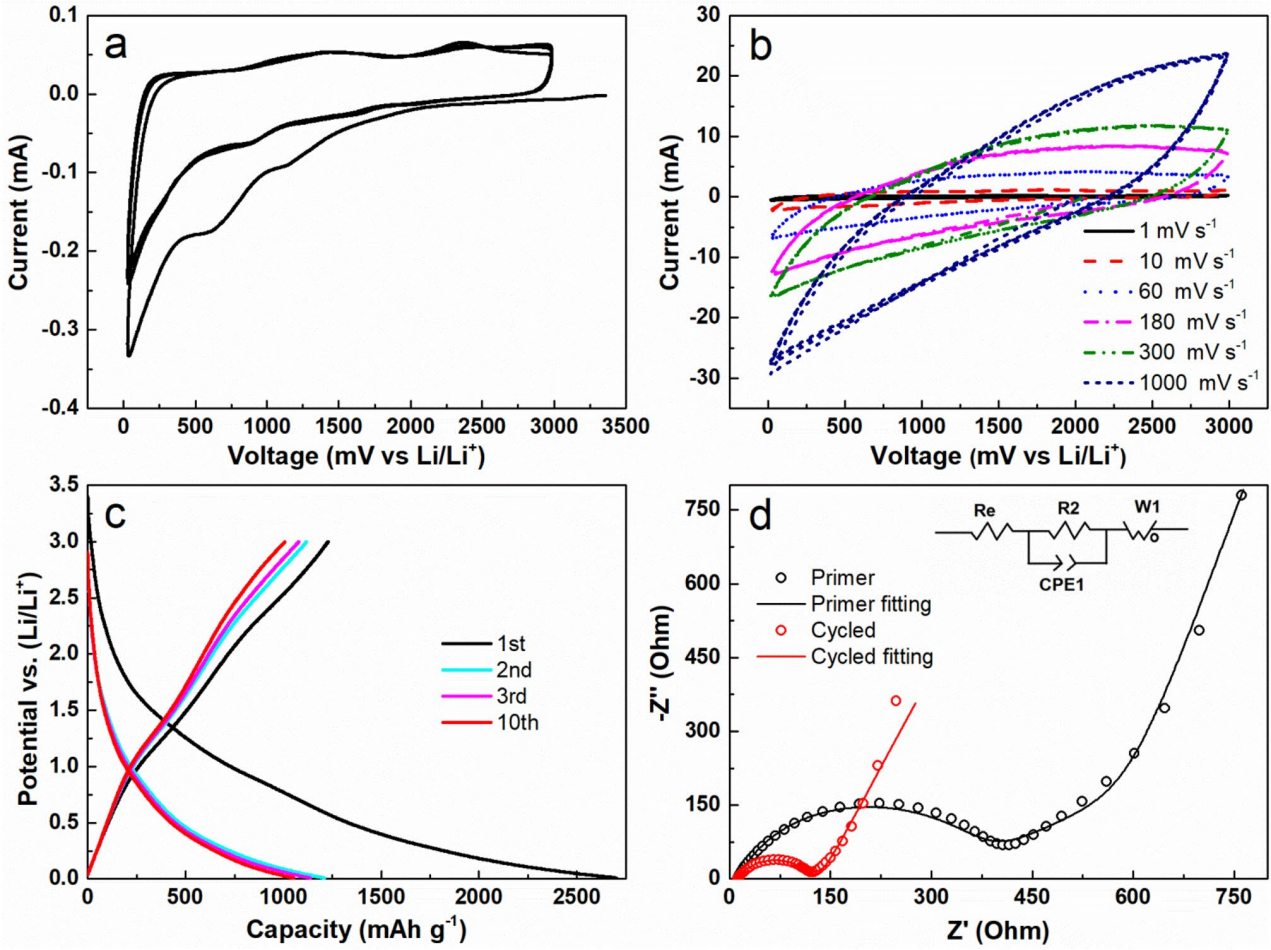
CV profiles of NBC@MWCNTs electrode in a potential range of 0.01 and 3 V: (**a**) at a scan rate of 0.2 mV s^−1^ of the initial three cycles; (**b**) at different scan rates from 1 to 1000 mV s^−1^; (**c**) Galvanostatic discharge/charge voltage profiles of different cycles at a current density of 0.1 A g^−1^ between 0.01 and 3 V for the NBC@MWCNTs electrode; (**d**) EIS of NBC@MWCNTs anode in a test window range from 100 kHz to 0.01 Hz before and after 10 electrochemical cycles for the cycling performance test at 0.1 A g^−1^. The inset is the equivalent circuit model of the studied system.

In order to systematically evaluate the lithium storage properties of the MWCNTs, Swagelok-type cells with two-electrode configuration were assembled in an argon-filled glovebox with lithium foil serves as counter/reference electrode. Figure 4a shows the initial three consecutive cyclic voltammetry (CV) cycles at a scan rate of 0.2 mV s^−1^ in the voltage range of 0.01–3.0 V versus Li/Li^+^. Compared with the subsequent two cycles, there exist two additional reduction peaks at approximately ~ 1.2 and ~ 0.7 V in the first cycle. These two peaks could be assigned to preliminary decomposition of the used electrolyte^35^. Furthermore, the reduction current in the first cycle below 1.3 V is significantly higher than that of the following two cycles. This extra current could be ascribed to the electrolyte being irreversibly reduced at low voltage to form a protective solid electrolyte interphase (SEI) layer^48^. In the subsequent cycles, these two peaks become weaker and slight shift to higher potential at around ~ 1.1 and ~ 1.7 V. There are two broad oxidation peaks in the following anodic processes at ~ 1.3 and ~ 2.3 V. The former peak is related to the partial decomposition of SEI components such as LiF, Li_2_CO_3_, and RCO_2_Li, and the latter peak is attributed to the reversible oxidation of N by other functional groups^37,49–51^. Accordingly, it can be seen that the following CV curves are quite similar after the first cycle, suggesting that both the formed SEI layer and the electrode material are highly stable.

Next, to study the electrochemical stability of the NBC@MWCNTs electrode, the same battery was further investigated with various scan rates increasing from 1 to 1000 mV s^−1^. As plotted in Fig. 4b, it can be observed that the LIB based on the NBC@MWCNTs anode has a very large discharge/charge current range. With the scan rate being increased step by step, the data still present good electrochemical stability. These positive results would indicate again that the fabricated NBC@MWCNTs electrode could supple a high-power output and fast charge, which is an ideal high performance anode for next-generation electric vehicles.

Figure 4c presents galvanostatic discharge/charge voltage curves of the as-obtained NBC@MWCNTs electrode for the first ten cycles at a current density of 0.1 A g^−1^ between 0.01 and 3 V. Surprisingly, at this current density, in the first cycle, an extreme high discharge capacity as high as 2701 mAh g^−1^ can be observed, which is calculated based on the total weight of the NBC@MWCNTs. In the following charge process, it still can maintain a high reversible capacity of about 1227 mAh g^−1^, which is more than 3.3 times higher than that of the theoretical maximum capacity for graphitic anode (LiC_6_, 372 mAh g^−1^). Accordingly, the irreversible capacity leads to an initial Coulombic efficiency of 45.4% (CE: defined by the charge capacity divided by the discharge capacity). In fact, the large irreversible capacity loss is an expected phenomenon for carbonaceous and other metal-oxides anodes in LIBs^37,52^. It is generally believed that the low efficiency value is probably because of the interfacial reactions, and the irreversible lithium ions insertion into some uncertain positions in the NBC@MWCNTs during the first discharge process^53^. However, this irreversible capacity loss could be much improved by prelithiation and/or some additional modifications in future experiments. In the following cycles, the capacity values of each discharge or charge are similar, indicating a stable lithiation and delithiation processes. Even after the tenth cycle, the charge capacity is still up to 1008 mAh g^−1^, corresponding to a high Coulombic efficiency of 95.8%. These similar features are consistent with the results that observed in CV curves.

To evaluate the effects of the NBC@MWCNTs electrochemical properties, subsequent electrochemical impedance spectroscopy (EIS) measurement were conducted on the NBC@MWCNTs electrode before cycling, and after tenth cycle, as plotted in Fig. 4d. The measured impedance data were analyzed by fitting to an equivalent circuit model (inset of Fig. 4d). The symbols denote the experimental data, while the continuous lines represent the fitted spectra. It is obvious to see that the charge transfer resistance (R_2_) changes from ~ 400 Ω of the fresh battery to ~ 120 Ω of the cycled battery based on the fitting results. This is commonly occurring in activity process, which is related to the electrochemical reactions of NBC@MWCNTs electrode with lithium ions, and other additional irreversible reactions^37^. This behaviour should be favourable for the transportation of lithium ions and electrons during repeated lithiation/delithiation processes, which agrees well with the results obtained from galvanostatic discharge/charge tests (Fig. 4c).

**Figure 5:**
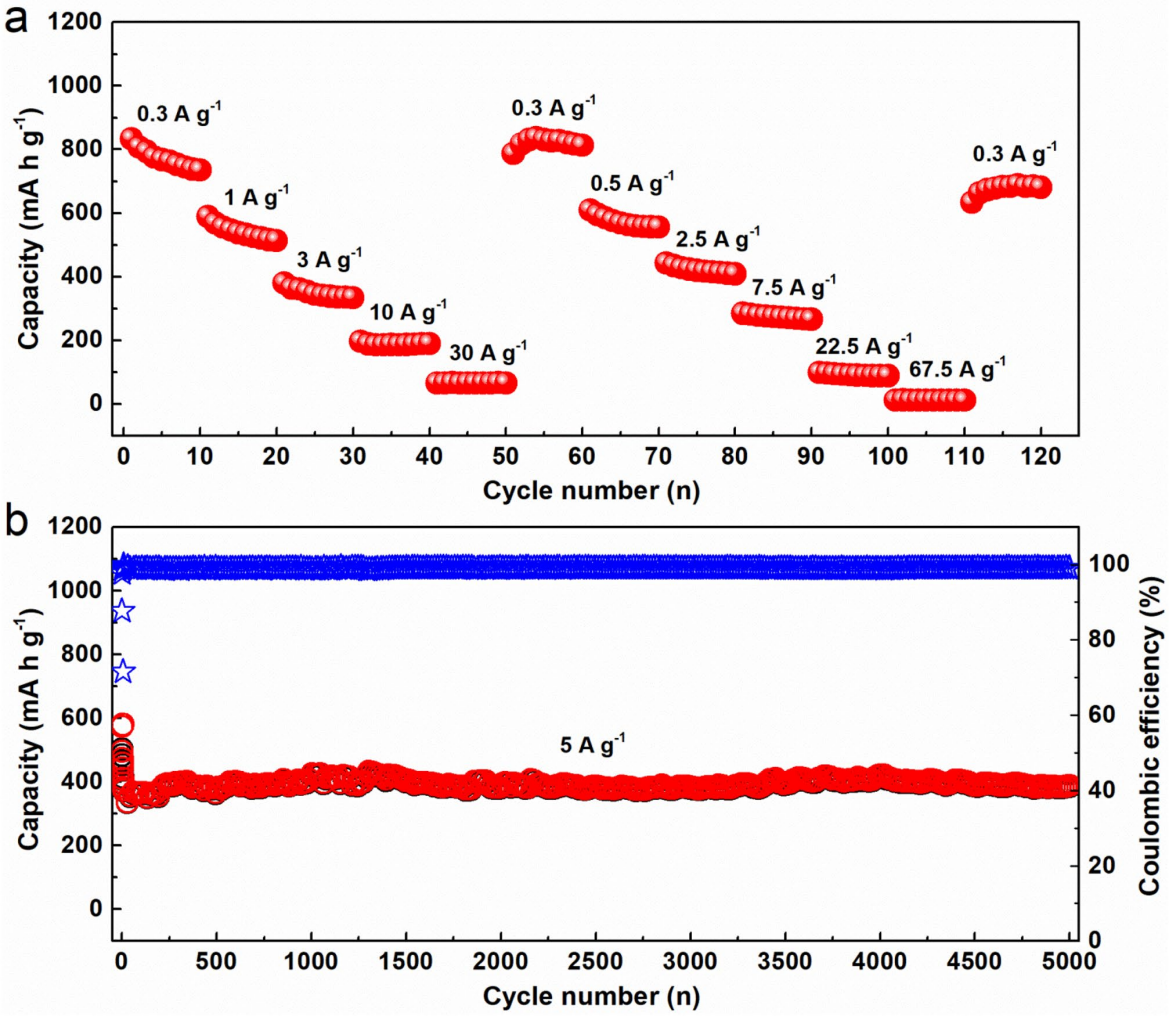
(**a**) Rate capability at various current densities from 0.3 to 67.5 A g^−1^, and (**b**) Cycling stability and Coulombic efficiency versus cycle number over a long life of 5000 cycles at a high current density of 5 A g^−1^.

The rate capability is an important parameter of LIBs for many applications that require fast discharge/charge rates. Herein, in order to confirm the high rate performance of the NBC@MWCNTs, the NBC@MWCNTs LIB was tested under different discharge/charge current densities from 0.3 to 67.5 A g^−1^ over 120 cycles. As shown in Fig. 5a, the battery was first tested from a small current density of 0.3 A g^−1^ to an extreme high current density of 30 A g^−1^ for each of 10 cycles. As a result, the battery could exhibit high capacity retention from ~ 870 to ~ 90 mAh g^−1^ and present a constant charge capacity in 1, 3, 10, and 30 A g^−1^ in the first test series. Moreover, the capacity could recover to a high capacity of 870 mAh g^−1^ when the current density is set back to 0.3 A g^−1^ after 60 cycles, showing highly flexible current test ability. This remarkable rate capability could be repeated again in the following high-rate experiment. It can be seen from the second test series, the battery still possess high lithium storage performance. It is noteworthy that, even after testing at an ultra-high current density of 67.5 A g^−1^, the electrode can still return to the original value when the current density suddenly decreases to 0.3 A g^−1^ after 120 cycles. This result demonstrates that the NBC@MWCNTs electrode has excellent high current or power output ability, which means it is a potential candidate to reduce charging time of rechargeable batteries to make it possible to promote the popularity of electric vehicles.

Furthermore, the ultra long term cycling stability of the NBC@MWCNTs electrode was also evaluated and the discharge/charge capacities, as well as Coulombic efficiency versus cycle number over 5000 cycles in the voltage range of 0.01–3 V at a high current density of 5 A g^−1^ are displayed in Fig. 5b. It should be mentioned that the measurement protocol was started with a small current density of 0.1 A g^−1^ for ten cycles to activate the anodic electrode thoroughly. As anticipated, the NBC@MWCNTs electrode also preforms unprecedented cycling stability, retaining high specific capacity of ~ 400 mAh g^−1^ after 5000 cycles with a superior average Coulombic efficiency of more than 99.2% at a high current density of 5 A g^−1^.

**Figure 6:**
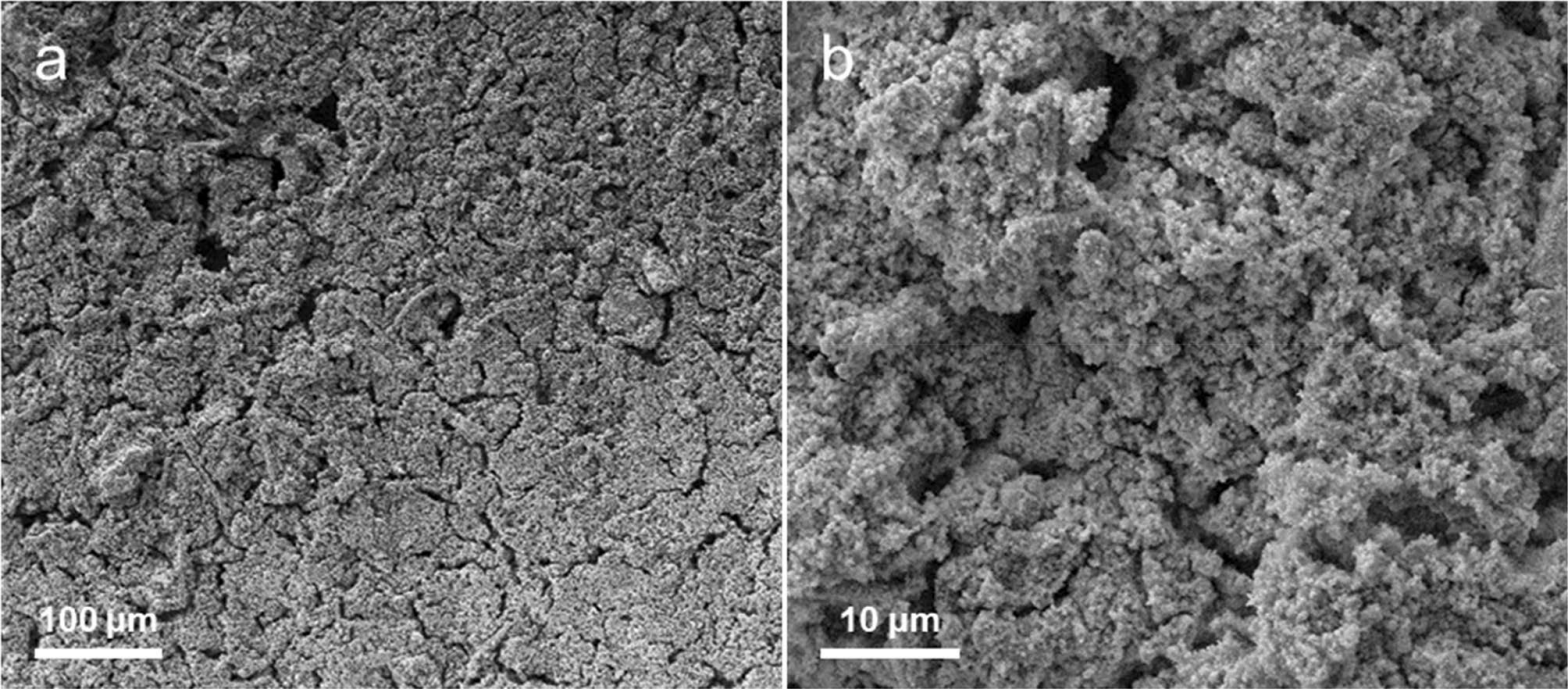
(**a**,**b**) SEM images at different magnifications of NBC@MWCNTs electrode at fully charged state after 5000 repeated electrochemical cycles under a constant current density of 5 A g^−1^.

To gain more insight into post-morphology of NBC@MWCNTs electrode, the cycled cell was manually disassembled and obtained cycled electrode was rinsed with propylene carbonate for further SEM observations. As evidenced in Fig. 6, it is interesting that the surface morphology of the NBC@MWCNTs electrode involves negligible changes even after 5000 discharge/charge cycles. More specifically, the as-prepared NBC@MWCNTs electrode still remains integrity and contains no large cracks, and contacts with the current collector tightly. These findings would account for the enhanced reaction kinetics and improved performance. In other words, the excellent electrochemical properties are therefore related to the good conductive paths for electrons, more lithium ions accessible sites introduced by N, B, and O doping, and good structural stability of the as-prepared electrodes.

In conclusion, highly porous N, B-doped carbon@MWCNTs were successfully prepared by pyrolysis of a thin layer of metal–organic framework complex coated on MWCNTs. When used as anode materials for LIBs, the fabricated NBC@MWCNTs electrode exhibits high stable capacity over an ultra-long cycling lifespan (5000 cycles) and superior rate capability even at very high current density (67.5 A g^−1^). Therefore, it is obvious that the NBC@MWCNTs could hold better electrochemical performance than previous reported carbon-based materials. Such impressive lithium storage properties could be ascribed to the synergistic coupling effect of the distinctive structural features, the reduced diffusion length of lithium ions, more active sites generated by doped atoms for lithium storage, as well as the enhancement of the electrode structural integrity. Our ongoing efforts are aligned to ascertain and mitigate the irreversible capacity losses in the first few cycles. Taken together, these results indicate that the N, B-doped carbon@MWCNTs materials may have great potential in future-generation LIBs for energy storage applications.

## Methods

### Materials preparation

All chemicals and materials were purchased from commercial suppliers and used as received without further purification. Typically, multiwall carbon nanotubes (MWCNTs, 773,840, Sigma-Aldrich) were chemically oxidized in nitric acid (65 wt%) for 48 h before use according to previously reported methods^54,55^. A certain amount of acid-oxidized MWCNTs (120 mg) was first dispersed in water/ethanol (v/v, 30 mL/100 mL) under ultrasonication for 2 h. Then, 1.562 g (100 mmol) 4,4′-bipyridine (denoted as Bpy) was dissolved in the above MWCNTs colloids under moderate stirring conditions, and diluted with deionized water to bring the volume to 1000 mL. The MWCNTs-Bpy solution was under stirring overnight, allowing the equilibrium adsorption of Bpy on the oxidized sites of MWCNTs. After this, 50 mL CuCl_2_·2H_2_O solution (10 mM) was directly added into the MWCNTs-Bpy solution under strong magnetic stirring to allow sufficient mixing and the products were formed in 30 min. Subsequently, the collection of the resultant products was centrifuged with the speed of ca. 4200 rpm for 12 min and washed for several times. After drying at 80 °C for 24 h, the obtained CNT-coordinated polymer composite could be obtained. The further treatment of the composite samples was referred to the method of salt template. In a typical process, the obtained composite polymer (1.2 g) was mixed and finely ground with salts mixtures (lithium chloride/zinc chloride/boron trioxide, 2.33 g/2.11 g/0.80 g). Then, the above mixtures were performed in a tube furnace at 900 °C for 3 h with a heating rate of 20 °C min^−1^ under the protection of argon atmosphere. Final hybrid materials were obtained after selectively removing the metal species with 4 M HNO_3_ solution (30 mL, immersing for 24 h) and subsequent intensive washing by deionized water and vacuum drying.

### Materials characterization

Scanning electron microscopy (SEM) and the corresponding elemental mapping analysis were determined by a scanning electron microscope (SEM, DSM982 Zeiss Germany). High-resolution transmission electron microscopy (TEM) was carried out using a Carl Zeiss Libra 200 Cs MC scanning (S) TEM operating at an accelerating voltage of 200 kV. Energy-dispersive X-ray spectroscopy (EDX) elemental mappings were acquired at STEM mode. The elements composition of the sample was characterized by X-ray photoelectron spectroscopy (XPS) PHI 5600 Survey with a standard Al-Kα source (1486.6 eV) operating at 350 W. Raman spectroscopy was collected by a Renishaw in Via Raman microscope system with 442 nm laser excitation. The laser beam was focused onto the sample by means of means of a 50 × objective lens and the laser power density was 0.4 mW µm^2^ at room temperature.

### Electrochemical measurements

All electrochemical measurements were studied at room temperature using two-electrode Swagelok-type cell configuration. The working anodes for testing were made of NBC@MWCNTs, carbon black, and polyvinylidene fluoride (PVDF) binder with a weight ratio of 70:20:10 in an N-methyl pyrrolidone (NMP). The mixed slurry was pasted onto a copper foil, and dried at 120 °C overnight in a vacuum oven. The sample was then assembled in Swagelok-type cell with using lithium foil as counter electrode and a solution of 1.0 M LiPF_6_ in ethylene carbonate/diethyl carbonate/dimethyl carbonate (EC/DEC/DMC; 1:1:1 vol.%, BASF) as electrolyte in an argon-filled glovebox (MBraun, Germany), in which has a low H_2_O and O_2_ level below 0.1 ppm. Whatman glass fiber filters (GF/D) were used as separators. The cyclic voltammetry (CV) and electrochemical impedance spectroscopy (EIS) characterizations were both performed using a Zahner IM6 (Zahner Elektrik, Germany) at room temperature. CV tests were studied with various scan rates from 0.2 to 1000 mV s^−1^ in the voltage range of 0.01–3.0 V versus Li/Li^+^. EIS measurements were carried out with the frequency from 100 kHz to 10 MHz with a fixed perturbation voltage of 5 mV, which was stabilized by 30 min of rest to reach a stable state about 1.6 V. The galvanostatic discharge/charge cycling tests of the assembled cells were performed between 0.01 and 3.0 V at various current densities from 0.1 to 67.5 A g^−1^ on an Arbin BT2000 system, based on the active electrode material corresponding specific capacity. The cycled half-cell was manually disassembled and the obtained cycled electrode was rinsed with propylene carbonate, which was further investigated by SEM analysis.
